# Fournier Gangrene Associated with Sodium-Glucose Cotransporter-2 Inhibitors: A Pharmacovigilance Study with Data from the U.S. FDA Adverse Event Reporting System

**DOI:** 10.1155/2020/3695101

**Published:** 2020-07-08

**Authors:** Yang Hu, Ziyu Bai, Yan Tang, Rongji Liu, Bin Zhao, Jian Gong, Dan Mei

**Affiliations:** ^1^Pharmacy Department, Peking Union Medical College Hospital, Peking Union Medical College, Chinese Academy of Medical Sciences, Beijing, China; ^2^School of Pharmacy, Tianjin University of Traditional Chinese Medicine, Tianjin, China; ^3^Research Group of Pharmacoepidemiology and Clinical Drug Evaluation, Shenyang Pharmaceutical University, Liaoning, China

## Abstract

**Background:**

The U.S. Food and Drug Administration (FDA) released a safety warning of Fournier gangrene (FG), a rare but serious adverse effect of sodium-glucose cotransporter-2 (SGLT2) inhibitors in August 2018. However, existing studies have focused mainly on individual FG case reports. Although several previous studies conducted reviews of cases, objective scientific analysis was not applied, and the prognosis data were inadequate.

**Objective:**

This study is aimed at presenting data supplementary to existing studies by analysing postmarketing adverse event reports in the FDA Adverse Events Reporting System (FAERS) database. Multiple statistical analysis methods were applied to evaluate the potential association between SGLT2 inhibitors and FG, thus providing reliable and professionalized medication usage recommendations for SGLT2 inhibitors in clinical practice.

**Methods:**

Disproportionality analysis and Bayesian analysis were applied for data mining among the suspected adverse event reports of FG associated with SGLT2 inhibitors recorded in the FAERS database during the period from January 2004 to September 2019.

**Results:**

There were 542 FG cases identified in the FAERS database in patients receiving SGLT2 inhibitors. Among all SGLT2 inhibitor therapies, empagliflozin was associated with the highest number of FG reports (232 in total), while empagliflozin plus metformin had the strongest association with FG occurrence with the reporting odds ratio (ROR 54.79, 95% two-sided CI 31.56 to 95.12) and proportional reporting ratio (PRR 53.36, *χ*^2^ 666.70). There were 391 patients who underwent initial or prolonged hospitalization (72.14%), and 26 patients died (4.81%). Three new FG cases caused by ertugliflozin were found in 2019.

**Conclusion:**

The analysis of SGLT2 inhibitor-associated FG reports in the FAERS database identified signals between the drug and adverse events of interest. It also provides comprehensive information on the characteristics of FG onset and prognosis. Clinicians should pay more attention to this rare but severe adverse event when prescribing SGLT2 inhibitors in clinical practice.

## 1. Introduction

Sodium-glucose cotransporter-2 (SGLT2) inhibitors are a relatively new drug category used for type 2 diabetes mellitus treatment in adults. They prohibit the normal functions of SGLT2, which are primarily expressed in the kidney, responsible for reabsorption of glucose from the glomerular filtration back into circulation, leading to urinary glucose excretion, thus lowering blood sugar. Unlike other oral antidiabetic drugs, SGLT2 inhibitors have no effects on endogenous glucose secretion and act independently of insulin production and pathways. This urinary glucose excretion also results in urinary volume increase and caloric loss [1]. SGLT2 inhibitors are recommended as second-line antidiabetics based on the American Diabetes Association (AMA) Standards of Medical Care in Diabetes 2019. They are the best choice for type 2 diabetes patients with atherosclerotic cardiovascular disease (ASCVD), heart failure (HF), or chronic kidney disease (CKD) [2].

However, it is believed that increased glucosuria can trigger urogenital tract infections and genital infections, which are the most common side effects of SGLT2 inhibitors [3, 4]. In addition, SGLT2i is also associated with a rare but life-threatening disease, the necrotizing fasciitis (NF) of the perineum, which is also referred to as Fournier's gangrene (FG). The FDA has released a warning about the occurrence of the FG that triggered by the usage of SGLT2i. It can affect the perianal, genitourinary, and perineal areas with the common symptoms of scrotal swelling, fever, and pain [5, 6]. In general, the mortality rate of FG can reach approximately 40% [7]. Urgent surgical debridement and broad-spectrum antibiotics are the key interventions. Also, approximately 20-70% of FG cases are associated with diabetes mellitus [5].

Although an increasing number of studies have aimed at exploring the potential association between FG and SGLT2 inhibitors, most of them were case reports or lacked scientific analysis. Thus, the objective of our study is to utilize multiple statistical analysis and data mining methods to analyse the possible association between different SGLT2 inhibitors and FG risks among spontaneous reports in the FAERS database. Furthermore, we present more comprehensive information on patient characterizations and prognosis outcomes as supplementary data to previous studies to provide advice on the clinical usage of SGLT2 inhibitors.

There are a large number of clinical trials, case reports, and other studies aimed at exploring the potential association between FG and SGLT2 inhibitors. Yet the overall number, follow-up, and size of trials are too low to draw liable conclusions, and patients enrolled in clinical trials cannot fully represent those who can receive a drug prescription in clinical practice, though the randomization procedure minimizes the possibility of bias [8–10]. While case reports are lack of ability to generalize. In contrary, the pharmacovigilance research with data from spontaneous reports is a valuable source of information to characterize the FG reports and identify potential risk factors [8]. Thus, the objective of our study is to utilize multiple statistical analysis and data mining methods to analyse the possible association between different SGLT2 inhibitors and FG risks among spontaneous reports in the FAERS database. Furthermore, we present more comprehensive information on patient characterizations and prognosis outcomes as supplementary data to previous studies to provide advice on the clinical usage of SGLT2 inhibitors.

## 2. Methods

### 2.1. Data Sources

We conducted a retrospective pharmacovigilance study that included all FG reports among patients treated with SGLT2 inhibitors from the first quarter of 2004 to the third quarter of 2019 in the U.S. FAERS database. This database was established by the FDA to support its postmarketing safety surveillance programme for drugs and therapeutic biologic products. It is a well-organized spontaneous reporting database that contains adverse events resulting from medication error reports, adverse event reports, and product quality complaints. The FAERS accepts global voluntary reports from healthcare professionals (physicians, pharmacists, nurses, and others), consumers (patients, family members, lawyers, and others), and drug companies.

A duplication process was then conducted, and other causes of FG among patients taking SGLT2 inhibitors were excluded. We screened 13,229,847 reports from the FAERS database and removed duplicated records according to the FDA's recommendations by selecting the latest FDA_DT when the CASEID and FDA_DT were the same. We ultimately included 11,115,435 reports for further analysis.

### 2.2. Adverse Event and Drug Identification

After literature review and summary of previous studies, we queried the FAERS to collect submitted FG reports in association with SGLT2 inhibitor treatment by applying the following preferred names from the Medical Dictionary for Regulatory Activities Terminology (MedDRA, version 22.1): fasciitis [10016228], fascial infection [10056515], Fournier's gangrene [10017068], necrotizing fasciitis [10028885], necrotizing fasciitis staphylococcal [10028887], necrotizing fasciitis streptococcal [10028888], necrotizing soft tissue infection [10076637], perineal abscess [10052457], perineal cellulitis [10078797], perineal infection [19966876], perineal necrosis [10076566], perineal operation [10062068], scrotal abscess [10049571], scrotal gangrene [10039748], vulval abscess [10047732], and vulval cellulitis [10047752]. The MICROMEDEX (Index Nominum) was used as a dictionary of SGLT2 inhibitors (Table 1).

### 2.3. Data Mining

To investigate the potential signals between the drug and the specific adverse event of interest, disproportionality analysis and Bayesian analysis were applied with the use of the reporting odds ratio (ROR), proportional reporting ratio (PRR), Bayesian confidence propagation neural network (BCPNN), and multi-item gamma Poisson shrinker (MGPS) algorithms. One of the four algorithms meet the criteria should be considered as a positive signal of FG.

The equations and criteria for the four algorithms above [11–19] are demonstrated in Table 2. The adverse events were collected as long as ≥1 index met the criteria.

^∗^ *a* is the number of reports containing both the suspected drug and the suspected adverse drug reaction; b is the number of reports containing the suspected adverse drug reaction with other medications (except the drug of interest); c is the number of reports containing the suspected drug with other adverse drug reactions (except the event of interest); d is the number of reports containing other medications and other adverse drug reactions. Abbreviations: ROR: reporting odds ratio; CI: confidence interval; N: number of co-occurrences; PRR: proportional reporting ratio; *χ*^2^: chi-squared; BCPNN: Bayesian confidence propagation neural network; IC: information component; IC025: lower limit of the 95% two-sided CI of the IC; MGPS: multi-item gamma Poisson shrinker; EBGM: empirical Bayesian geometric mean; EBGM05: lower 90% one-sided CI of the EBGM.

### 2.4. Statistical Analysis

Descriptive analysis was applied to summarize and present the clinical characteristics of the patients in FG reports. The time to onset of FG induced by different SGLT2 inhibitor therapies was compared by nonparametric tests (the Mann-Whitney test for dichotomous variables and the Kruskal-Wallis test for >2 subgroups of respondents) since the data were not normally distributed. Pearson's chi-square test or Fisher's exact test was utilized to compare the mortality rate of FG in patients receiving different SGLT2 inhibitors. Statistical significance was declared at *P* < 0.05 with 95% confidence intervals (CIs). The data mining and statistical analysis were assessed by SAS version 9.4 (SAS Institute Inc., Cary, NC, USA).

## 3. Results

In total, there were 42,531 FG adverse events retrieved from the FAERS database during the period from 2004q1 to 2019q3, in which 5562 were in correlation with the usage of SGLT2 inhibitor therapies. After excluding duplications, 542 reports met our criteria and were identified. The detailed information of the reports is shown in Table 3. As for the basic characteristics of the patients, the FG occurrence in males (64.3%) was significantly higher than that in females (27.9%), with 7.8% of unknown gender. Except for two patients aged <18 years, all patients were adults, with an average age of 55.6 years (range from 0 to 87 years). For the reports, approximately two-thirds of overall reports were submitted by healthcare professionals, with the majority reported from North America. The number of FG reports fluctuated between 2014 and 2017 and then experienced a significant rise from 103 to 407 reports in 2018 and 2019, respectively. Initial or prolonged hospitalization was the most common outcome event. The FG onset time in patients received SGLT2 inhibitors as single antidiabetic treatment was ranged from 0 to 1365 days, while in patients combined SGLT2 inhibitors with other glucose-lowering medication (GLM) therapies, the range was 1696 days.

A total of 232 FG adverse event reports were related to empagliflozin, followed by canagliflozin, with 199 reports (Table 4). The disproportionality analysis and Bayesian analysis indicated that empagliflozin had the strongest potential associations with FG occurrence, with ROR (46.70, 95% two-sided CI 40.61 to 53.70), PRR (45.70, *χ*^2^ 8798.23), IC (5.46, IC025 4.75), and EBGM (44.02, EBGM05 39.16) values. Additionally, 26 patients died. Three FG cases were newly reported related to the use of ertugliflozin. No FG cases caused by dapagliflozin/saxagliptin, ertugliflozin/metformin, ertugliflozin/sitagliptin, ipragliflozin, luseogliflozin, or tofogliflozin were reported in the FAERS; the last three have been approved in Japan.

## 4. Discussion

To our knowledge, this study involved comprehensive information (relative to previous studies) of patients, reporting SGLT2 inhibitor usage and prognosis in spontaneous reports with the longest time interval in the FAERS database. The timing of FG onset and the mortality rate were assessed as well.

Overall, 542 SGLT2 inhibitor-associated FG case reports were identified in the FAERS database from January 2004 to September 2019, while the total report number in Table 3 was 534, which resulted in inadequate information presented in some of the reports. Based on our research, the average age was 55.6 years. Two patients were <18 years old, which was considered off-label use according to the SGLT2 inhibitor instructions. These data were consistent with the study conducted by Fadini et al. [20], though the sample size was relatively small, with 31 men and 11 women and a mean age of 58.9 years. We found that FG was more common in men (64.3%) than in women (27.9%). It seems that adult men with type 2 diabetes are more susceptible to FG. The reasons for the relatively lower occurrence of FG in women could relate to the better drainage of the perineal region by vaginal secretions in females [21], as well as physicians' lack of recognition of FG symptoms in women [22].

Based on the literature review, few studies have performed analyses on report characteristics in detail. However, it is essential to analyse the voluntary reports in various aspects to gain an overall understanding of the changes in the FG occurrence and the tendency for a particular reporting. The FAERS database accepts the suspected adverse events from consumers, healthcare professionals, and manufacturers. In our study, 319 (58.86%) FG reports were submitted by healthcare professionals, of which 43.00% were reported by physicians. Consumers (including two lawyers) were responsible for 193 (35.61%) of the overall reports. We also found that the number of submitted reports experienced a significant rise from 1 case in 2014 to 407 additional cases in 2019. Fadini et al. showed that the FG report rate increased fourfold in 2018q4 (with 51 new cases) since the FDA warning. This dramatic increase might be due to the popularity of the usage of SGLT2 inhibitors rising rapidly worldwide along with the increased needs of patients with a high occurrence of diabetes [20]. The awareness of SGLT2 inhibitor safety among patients was strengthened as well [23]. In addition, this may reflect that SGLT2 inhibitors were being used in progressively fewer selected populations, with FG-predisposing factors such as an impaired immune system.

**Figure 1:**
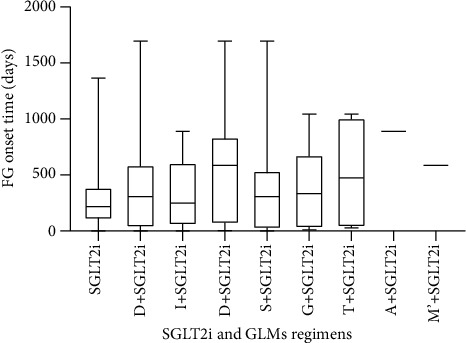
The onset time of FG in patients receiving SGLT2 inhibitors alone and with other glucose-lowering medications. Abbreviations: GLMs: glucose-lowering medications; SGLT2i: sodium-glucose cotransporter-2 inhibitors (canagliflozin, dapagliflozin, empagliflozin, ertugliflozin); M: metformin; I: insulin; D: dipeptidyl peptidase-4 inhibitors (alogliptin, sitagliptin, linagliptin, saxagliptin, teneligliptin); S: sulfonylurea (repaglinide, gliclazide, glimepiride, glipizide, glibenclamide); G: glucagon-like peptide-1 receptor agonists (exenatide, dulaglutide, liraglutide, semaglutide); T: thiazolidinediones (pioglitazone); alpha-glucosidase inhibitors (acrbose); M′: meglitinides (repaglinide).

There are 1019 concomitant drugs in total with the usage of SGLT2 inhibitors taken by patients with FG, which includes 316 (31.00%) antidiabetic medicines. Medications used to treat cardiovascular disorders were the most common concomitant drugs in this study, with the percentage of 34.84%. Metformin and insulin treatments are the major concomitant antidiabetic drugs with a proportion of 34.81% and 31.01%, respectively (Table 5). The onset time of FG with patients under SGLT2 inhibitors as single treatment as well as SGLT2 inhibitors concomitant with other glucose-lowering medication (GLM) therapies is shown in Figure 1. Overall, 449 cases were founded in the FAERS database with the information of FG occurrence time. After removing the cases with incomplete or incorrect information, 134 cases were qualified, in which 71 patients were taking the SGLT2 inhibitors alone and 52 patients were on the other antidiabetic treatments along with the use of the SGLT2 inhibitors. The mean FG onset time of SGLT2 inhibitors as the single treatment for patients was 293 days, with a range of 0 to 1365 days. The metformin was the most commonly used antidiabetic drug compared to other antihyperglycemic drugs; the average onset time was 373 days (range from 0 to 1696 days) when used in combination with SGLT2 inhibitors. We consider conducting further scientific analysis between the onset time of FG associated with different drug therapies; however, the data was too small to make a valuable comparison.

As a single report could contain more than one outcome, 826 clinical outcomes were collected. After removing duplicates, 534 valid reports were identified from the FAERS database. Among these, initial or prolonged hospitalization was the most common clinical outcome among all SGLT2 inhibitor treatments. A study indicated a similar result, with 53 patients hospitalized (initial or prolonged) in all 55 cases [24]. Approximately one-fifth of patients were in life-threatening status. Another essential finding was that no FG cases reported ertugliflozin as a primary suspect in any existing study, which might be due to its shorter marketing time as a newly approved SGLT2 inhibitor [20, 24]. Nevertheless, 3 new FG cases developed because of the usage of ertugliflozin in 2019. This was also the drug with the shortest FG average onset time (223 days) among all SGLT2 inhibitor regimens. Therefore, the FG adverse event could be seen as a class effect of all SGLT2 inhibitor therapies. These data can serve as supplementary information for existing SGLT2 inhibitor-associated FG studies. Although no FG cases associated with ertugliflozin/metformin or ertugliflozin/sitagliptin have been reported to date, attention should be paid by healthcare professionals when using them in clinical practice.

Notably, one case with type 1 diabetes mellitus was 53 years old and received Farxiga® as the antidiabetic treatment. However, there was no record of the exact FG onset time of this patient, and the outcome was classified as “other serious (important medical event).” The package inserts of Farxiga® clearly note that this drug is not recommended for patients with type 1 diabetes mellitus [25]. The FDA has not approved any SGLT2 inhibitors for this particular indication, although several clinical trials are ongoing. Joury et al. and Wu et al. supposed that SGLT2 inhibitors can deteriorate ketogenesis and contribute to DKA, particularly in patients with type 1 diabetes [7, 26]. Hence, the benefit-risk relationship between the usage of SGLT2 inhibitors and type 1 diabetes mellitus still needs to be further assessed. Whether this off-label usage of SGLT2 inhibitors was reasonable may need more evidence.

To investigate the potential association between different SGLT2 inhibitor therapies and FG occurrence, disproportionality analysis and Bayesian analysis were applied (Table 4). Empagliflozin was associated with the highest number of FG reports, 232 in total, followed by canagliflozin and dapagliflozin, with 199 and 108 reports, respectively. The results show that empagliflozin had the strongest association with the onset of FG, with higher ROR, PRR, IC, and EBGM values than any other SGLT2 inhibitors. There was no previous study applying disproportionality analysis and Bayesian analysis in SGLT2 inhibitors; thus, these data could be utilized as compensatory information. It is widely known that diabetes is a risk factor for FG, and some studies have investigated the association between diabetes, FG, and SGLT2 inhibitor therapies. They confirmed that the association with FG was more specific for SGLT2 inhibitor therapies than diabetes [20]. Furthermore, our previous research suggested that dapagliflozin treatment was associated with an increased risk of developing genital infections [4]. We therefore advocate conducting more drug pharmacovigilance programmes for SGLT2 inhibitors so that the awareness of those serious adverse effects among patients and clinicians will be enhanced, especially when choosing a treatment.

A notoriety bias indeed exists that is a selection bias in which a case has a greater chance of being reported if the subject is exposed to the studied factor known to cause, thought to cause, or likely to cause the event of interest. Thus, it could partly explain the increasing FG reports numbers after FDA warning in August 2018. It is a common issue that happens in lots of drugs. A study conducted by Neha et al. confirms that notoriety bias does not lead to overreporting in the FAERS database, and the overall disproportionality in signal estimates is not changed by the safety alert [10].

However, this study does have some limitations. First, for the FAERS database, a major concern was underreporting of adverse events, although the “consumer-friendly” reporting form FDA3500B was developed [27]. The data mining approach cannot compensate for this limitation. Moreover, adverse event reports might be associated with false and incomplete information since not all reporters have expertise in pharmacovigilance activities, leading to the quality of the reports being different. The relationship between the occurrence of the reported event and the product was uncertain, as the FDA did not require reporters to provide evidence on the causal relationship between a product and the event. Also, the spontaneous reporting system cannot quantify the adverse reaction signals of FG on the basis of the total number of adverse reactions. It also cannot calculate the exact incidence of SGLT2 inhibitor-associated FG, since the association between a drug and an adverse drug reaction is confounded by concomitant drugs and comorbidities [28]. Detailed information is also needed from clinical follow-up and other surveys to verify our data mining hypotheses. Finally, the data mining approach was not sufficient to prove a causal relationship between a drug and an adverse event, though it can perform signal detection.

## 5. Conclusions

In summary, our study applied multiple statistical analysis approaches to identify the signals of FG caused by different SGLT2 inhibitors in the FAERS database. This study also presented comprehensive prognosis data as supplementary information to existing studies. Empagliflozin had the greatest association with the occurrence of FG and the highest mortality rate. Even the new SGLT2 inhibitor member could also induce FG. Moreover, the total number of FG reports in the FAERS database has increased significantly. Since it is expected that the number of SGLT2 inhibitor users will continually increase in the future, paying more attention to severe adverse events is very important even if they are extremely rare.

Thus, the drug pharmacovigilance programme is necessary and essential to monitor FG in diabetic adults receiving SGLT2 inhibitors to evaluate drug safety concerns, facilitating the FDA to improve product safety and protect public health. Further pharmacoepidemiological studies are needed to provide more reliable evidence for the use of SGLT2 inhibitors in clinical practice.

## Figures and Tables

**Table 1:** Summary of the generic name and brand name of SGLT2 inhibitors based on MICROMEDEX®.

Generic name	Brand name
Canagliflozin	Canaglu®, Invokana®
Canagliflozin/metformin hydrochloride	Invokamet®, Invokamet XR®, Vokanamet®
Dapagliflozin propanediol	Edistride®, Farxiga®, Forxiga®, Forziga®
Dapagliflozin propanediol/metformin hydrochloride	Ebymect®, Xigduo®, Xigduo XR®
Dapagliflozin propanediol/saxagliptin	Qtern®
Empagliflozin	Jardiance®
Empagliflozin/linagliptin	Glyxamb®
Empagliflozin/metformin hydrochloride	Jardiamet®, Jardiance duo®, Synjardy®, Synjardy XR®
Ertugliflozin	Steglatro®
Ertugliflozin/metformin hydrochloride	Segluromet®
Ertugliflozin/sitagliptin	Steglujan®
Ipragliflozin	Suglat®
Luseogliflozin	Lusefi®
Tofogliflozin	Apleway®, Deberza®

**Table 2:** Summary of major algorithms used for signal detection.

Algorithms	Equation^∗^	Criteria
ROR	$\text{ROR}=\frac{a/b}{c/d}$	95% CI>1, *N* ≥ 2

95%CI = *e*^^ln(ROR)±1.96(1/*a* + 1/*b* + 1/*c* + 1/*d*)^^0.5^^

PRR	$\text{PRR}=\frac{\left(a\right)/\left(a+c\right)}{\left(b\right)/\left(b+d\right)}\;$	PRR ≥ 2, *χ*^2^ ≥ 4, *N* ≥ 3

*χ* ^2^ = ∑(*O* − *E*)2/*E*, (*O* = *a*, *E* = (*a* + *b*)(*a* + *c*) /(*a* + *b* + *c* + *d*))

BCPNN	IC = log_2_(*a*(*a* + *b* + *c* + *d*)/(*a* + *c*)(*a* + *b*))	IC025 > 0

IC025 = *e*^^ln(IC)−1.96(1/*a* + 1/*b* + 1/*c* + 1/*d*)^^0.5^^

MGPS	EBGM = *a*(*a* + *b* + *c* + *d*)/(*a* + *c*)(*a* + *b*)	EBGM05 > 2, *N* > 0

EBGM05 = *e*^ln(EBGM)−1.64(1/*a* + 1/*b* + 1/*c* + 1/*d*)^0.5^^

**Table 3:** Detailed information on FG reports.

	Canagliflozin (*n*)	Dapagliflozin (*n*)	Empagliflozin (*n*)	Ertugliflozin (*n*)	Total (*n*, %)
Age of patient					
<18	1	1	0	0	
18-64	148	55	100	2	
>64	25	14	47	0	
Average (years)	56	57	57	51	
Gender of patient					
Male	130 (65.33%)	69 (63.89%)	146 (63.20%)	3 (100.00%)	
Female	58 (29.15%)	29 (26.85%)	64 (27.71%)	0 (-)	
Unknown	11 (5.53%)	10 (9.26%)	21 (9.09%)	0 (-)	
Average weight of patient (kg)	98	112	109	180	
Reporter					
Healthcare professionals	53	73	191	2	319 (58.86%)
Consumers	144	8	40	1	193 (35.61%)
Unknown	2	27	1	0	30 (5.54%)
Reported year					
2019	153	68	183	3	407 (75.09%)
2018	31	27	45	0	103 (19.00%)
2017	2	1	1	0	4 (0.74%)
2016	2	7	1	0	10 (1.85%)
2015	10	4	1	0	15 (2.77%)
2014	0	1	0	0	1 (0.18%)
Unknown	1	0	1	0	2 (0.36%)
Area					
North America	178	51	127	2	358 (66.05%)
Europe	17	40	65	0	122 (22.51%)
Asian	4	11	21	0	36 (6.64%)
Oceania	0	2	13	1	16 (2.95%)
South America	0	4	4	0	8 (1.48%)
Africa	0	0	1	0	1 (0.18%)
Unknown	0	0	1	0	1 (0.18%)
Outcome events (*n*, %)					
Hospitalization-initial or prolonged	172 (86.43%)	76 (72.38%)	141 (72.38%)	2 (66.67%)	
Other serious (important medical event)	55 (27.64%)	54 (51.43%)	133 (51.43%)	1 (33.33%)	
Life-threatening	30 (15.08%)	24 (22.86%)	50 (22.86%)	0 (0.00)	
Disability	28 (14.07%)	8 (7.62%)	19 (7.62%)	0 (0.00)	
Death	7 (3.52%)	7 (6.67%)	12 (5.29%)	0 (0.00)	
Required intervention to prevent permanent impairment/damage	3 (1.51%)	0 (0.00)	4 (1.76%)	0 (0.00)	
Total report number	199	105	227	3	

Note: no reports of ipragliflozin, luseogliflozin, or tofogliflozin.

**Table 4:** The association between different SGLT2 inhibitors and FG occurrence.

Drug	*N*	ROR	PRR	IC	EBGM
		(95% two-sided CI)	(*χ*^2^)	(IC025)	(EBGM05)
Canagliflozin	190	18.76 (16.22, 21.70)	18.60 (3058.02)	4.17 (3.61)	18.00 (15.94)
Canagliflozin/metformin	9	20.12 (10.43, 38.81)	19.93 (161.61)	4.31 (2.24)	19.90 (11.48)
Dapagliflozin	96	22.89 (18.68, 28.04)	22.64 (1952.68)	4.48 (3.65)	22.27 (18.79)
Dapagliflozin/saxagliptin	0	—	—	—	—
Dapagliflozin/metformin	12	34.86 (19.69, 61.73)	34.28 (387.09)	5.10 (2.88)	34.21 (21.21)
Empagliflozin	209	46.70 (40.61, 53.70)	45.70 (8798.23)	5.46 (4.75)	44.02 (39.16)
Empagliflozin/linagliptin	10	23.82 (12.76, 44.46)	23.55 (215.66)	4.56 (2.44)	23.51 (13.95)
Empagliflozin/metformin	13	54.79 (31.56, 95.12)	53.36 (666.70)	5.73 (3.30)	53.24 (33.56)
Ertugliflozin	3	18.68 (5.99, 58.24)	18.51 (49.70)	4.21 (1.35)	18.50 (7.15)
Ertugliflozin/metformin	0	—	—	—	—
Ertugliflozin/sitagliptin	0	—	—	—	—
Ipragliflozin	0	—	—	—	—
Luseogliflozin	0	—	—	—	—
Tofogliflozin	0	—	—	—	—

**Table 5:** All concomitant drugs and concomitant antidiabetic drugs received by FG patients under SGLT2 inhibitor therapy.

All concomitant drugs	*n* (%)	Concomitant antidiabetic drugs	*n* (%)
Cardiovascular system	355 (34.84%)	Metformin	110 (34.81%)
Endocrine system	329 (32.29%)	Insulin	98 (31.01%)
Nervous system	61 (5.99%)	Dipeptidyl peptidase-4 inhibitors	54 (17.09%)
Gastrointestinal system	60 (5.89%)	Sulfonylurea	37 (11.71%)
Blood and nutrition	48 (4.71%)	Thiazolidinediones	8 (2.53%)
Anti-inflammatory and analgesic	36 (3.53%)	Combination therapy	5 (1.58%)
Respiratory system	29 (2.85%)	Meglitinides	2 (0.63%)
Infection	22 (2.16%)	Alpha-glucosidase inhibitors	1 (0.32%)
Immune system and malignant disease	21 (2.06%)	Other	1 (0.32%)
Controlled drug	18 (1.77%)	Total	316
Genitourinary system	18 (1.77%)		
Others	17 (1.67%)		
Skin	4 (0.39%)		
Musculoskeletal system	1 (0.10%)		
Total	1019		

## Data Availability

The data that support the findings of this study are available from the corresponding authors upon reasonable request (zhaobin@pumch.cn).
